# De novo and inherited loss-of-function variants of ATP2B2 are associated with rapidly progressive hearing impairment

**DOI:** 10.1007/s00439-018-1965-1

**Published:** 2018-12-08

**Authors:** Jeroen J. Smits, Jaap Oostrik, Andy J. Beynon, Sarina G. Kant, Pia A. M. de Koning Gans, Liselotte J. C. Rotteveel, Jolien S. Klein Wassink-Ruiter, Rolien H. Free, Saskia M. Maas, Jiddeke van de Kamp, Paul Merkus, DOOFNL Consortium, Wouter Koole, Ilse Feenstra, Ronald J. C. Admiraal, Cornelis P. Lanting, Margit Schraders, Helger G. Yntema, Ronald J. E. Pennings, Hannie Kremer

**Affiliations:** 1 Hearing and Genes, Department of Otorhinolaryngology, Head and Neck Surgery, Radboud University Medical Center, Nijmegen, The Netherlands; 2 Donders Institute for Brain, Cognition and Behaviour, Radboud University Medical Center, Nijmegen, The Netherlands; 3 Department of Clinical Genetics, Leiden University Medical Center, Leiden, The Netherlands; 4 Department of Otorhinolaryngology, Head and Neck Surgery, LUMC, Leiden, The Netherlands; 5 Department of Clinical Genetics, University Medical Center Groningen, Groningen, The Netherlands; 6 Department of Otorhinolaryngology, Head and Neck Surgery, University Medical Center Groningen, Groningen, The Netherlands; 7 Department of Clinical Genetics, Amsterdam UMC, Vrije Universiteit Amsterdam, Amsterdam, The Netherlands; 8 Department of Otolaryngology, Head and Neck Surgery, Ear and Hearing, Amsterdam Public Health Research Institute, Amsterdam UMC, Vrije Universiteit Amsterdam, Amsterdam, The Netherlands; 9 Hearing and Genes, Department of Human Genetics, Radboud University Medical Center, Internal postal code 855, P.O. Box 9101, 6500 HB Nijmegen, The Netherlands

## Abstract

*ATP2B2* encodes the PMCA2 Ca^2+^ pump that plays an important role in maintaining ion homeostasis in hair cells among others by extrusion of Ca^2+^ from the stereocilia to the endolymph. Several mouse models have been described for this gene; mice heterozygous for loss-of-function defects display a rapidly progressive high-frequency hearing impairment. Up to now *ATP2B2* has only been reported as a modifier, or in a digenic mechanism with *CDH23* for hearing impairment in humans. Whole exome sequencing in hearing impaired index cases of Dutch and Polish origins revealed five novel heterozygous (predicted to be) loss-of-function variants of *ATP2B2*. Two variants, c.1963G>T (p.Glu655*) and c.955delG (p.Ala319fs), occurred de novo. Three variants c.397+1G>A (p.?), c.1998C>A (p.Cys666*), and c.2329C>T (p.Arg777*), were identified in families with an autosomal dominant inheritance pattern of hearing impairment. After normal newborn hearing screening, a rapidly progressive high-frequency hearing impairment was diagnosed at the age of about 3–6 years. Subjects had no balance complaints and vestibular testing did not yield abnormalities. There was no evidence for retrocochlear pathology or structural inner ear abnormalities. Although a digenic inheritance pattern of hearing impairment has been reported for heterozygous missense variants of *ATP2B2* and *CDH23*, our findings indicate a monogenic cause of hearing impairment in cases with loss-of-function variants of *ATP2B2*.

**Electronic supplementary material:**

The online version of this article (10.1007/s00439-018-1965-1) contains supplementary material, which is available to authorized users.

## Introduction

Hearing impairment (HI) is an important cause of social burden worldwide (WHO 2008). The reported incidence in early childhood varies from 1 to 3/1000 (Wroblewska-Seniuk et al. 2017). It is estimated that early onset sensorineural HI (SNHI) is hereditary in about 50% of the cases and in 20% of the cases nonsyndromic SNHI is inherited in an autosomal dominant pattern (Smith et al. 2005). Until now, more than 60 loci and 37 genes have been identified for autosomal dominant nonsyndromic SNHI (Hereditary Hearing loss Homepage). Still, for many subjects with congenital or childhood-onset HI, a genetic diagnosis cannot be provided (Zazo Seco et al. 2017). For several of the genes (recently) described to be associated with early onset HI, defects have so far been found in only a few or one single family. To confirm the association of such genes with HI, additional supportive data are required. Mouse models have contributed such evidence for several genes and have been of great significance by providing candidate genes for HI in humans (Brown et al. 2008; Friedman et al. 2007; Wesdorp et al. 2018).

The plasma membrane Ca^2+^ ATPase 2 **(**PMCA2), encoded by *Atp2b2*, has already been known for 2 decades to be essential for hearing and balance in mouse (e.g. the *deafwaddler* mouse) (Street et al. 1998). It is one of the two PMCAs with a tissue-specific expression; PMCA2 is mainly expressed in the inner ear, the cerebellum and the mammary gland. Alternative splicing of *Atp2b2* transcripts results in several PMCA2 isoforms, e.g. PMCA2 w/a, or z/a [for review (Strehler and Zacharias 2001)]. Isoform PMCA2 w/a is the major PMCA2 isoform in the organ of Corti and is encoded by a transcript with three alternatively spliced exons at the first alternative splice site, site A (Grati et al. 2006; Hill et al. 2006). Given the structural and functional homology of the inner ear in mice, rat and human, PMCA2 was anticipated to be critical for hearing in humans as well, and indications for that have already been reported. A hypofunctional variant of PMCA2 (p.Val586Met) was found to aggravate HI in a family with a homozygous *CDH23* variant (Schultz et al. 2005). Moreover, digenic inheritance of SNHI was suggested in an isolated case with heterozygous missense variants in both *ATP2B2* and *CDH23* (Ficarella et al. 2007). Furthermore, SNHI in cases with the 3p deletion syndrome (MIM 613792) was suggested to be correlated with haploinsufficiency of *ATP2B2* (McCullough et al. 2007). A heterozygous missense variant in *ATP2B2* has recently been associated with ataxia but without HI; the identified variant functionally affected a PMCA2 isoform that is expressed in the cerebellum but not prominently in the inner ear (Vicario et al. 2018). Finally, two de novo truncating variants in *ATP2B2* have been reported in a study on autism (Takata et al. 2018). These studies, however, do not provide evidence for a causative association of *ATP2B2* with monogenic SNHI.

Here, we report four different heterozygous truncating variants and one canonical splice site variant in *ATP2B2* in families with nonsyndromic progressive SNHI. In two of the families, the variants are de novo. All variants affect exons, or their splice sites, that encode the ortholog of the rat PMCA2 w/a isoform. This isoform is highly abundant in stereocilia of outer hair cells (OHC) and to a lesser extent at the apical surface of inner hair cells (IHC) (Hill et al. 2006).

## Materials and methods

### Clinical examination

This study was approved by the medical ethics committee of the Radboud University Medical Center in Nijmegen, the Netherlands and was carried out according to the Declaration of Helsinki. Written informed consent was obtained from all participants or their legal representatives. Medical history was taken from all participants, with special attention paid to (acquired) HI and vestibular symptoms. Affected individuals underwent general ear, nose and throat (ENT) examinations in the Radboud University Medical Center or findings of ENT examination were taken from their medical history. Pure tone audiometry (PTA) was performed according to current standards in a sound-attenuating booth, where air conduction hearing thresholds for the frequencies from 250 Hz to 8 kHz were measured. Bone conduction thresholds were measured for the frequencies 500 Hz–8 kHz. The age- and gender-specific 95th percentile thresholds for individual hearing levels for each frequency were derived from the International Organisation for Standardization; ISO 7029:2017. Individuals were considered affected when pure tone thresholds for at least three frequencies were below the 95th percentile (P95) for the better hearing ear. The GENDEAF guidelines for the description of audiological data were applied in this study (Mazzoli et al. 2003). GraphPad Prism 6.0 (GraphPad, San Diego, CA, USA) was used to calculate an age-related typical audiogram (ARTA), based on linear regression, for the ages of 10–70 years as described (Huygen et al. 2003). Speech perception was assessed in a sound-attenuating booth with standard monosyllabic consonant–vowel–consonant Dutch word lists (Bosman and Smoorenburg 1995). Otoacoustic emissions and click-evoked brainstem-evoked response audiometric (BERA) measurements were performed according to current standards. In the Netherlands, the first step of newborn hearing screening is carried out by the detection of transient evoked otoacoustic emissions (TEOAE) (van der Ploeg et al. 2012). If applicable, results of newborn hearing screening were assessed by hetero-anamnesis of parents. Vestibular function was assessed by electronystagmography (ENG), caloric irrigation testing, rotary chair stimulation and video head impulse tests (vHIT), as described previously (Oonk et al. 2015). Additionally, cervical and ocular vestibular-evoked myogenic potentials (cVEMP/oVEMP) were measured to assess saccular and utricular function, respectively, as described (Papathanasiou et al. 2014; Vanspauwen et al. 2017). When responses were seen at ≤ 100 dB hearing level in cVEMP testing, saccular function was considered as present, otherwise absent (Papathanasiou et al. 2014). For oVEMPs, this normal value is ≤ 140 dB force level (Vanspauwen et al. 2017).

Subjects V.2 of family W18-0139 and I.1 of family W17-0883 were not able to participate in clinical evaluations, but retrospective data were analyzed. Subjects IV.3, IV.4 and V.1 of family W18-0139 did not consent for the study.

### Genetic analyses

DNA was extracted from peripheral blood samples according to standard procedures. Exomes were enriched with the Agilent SureSelectXT Human AllExon v4 or v5 kit (Santa Clara, CA, USA) and sequencing was performed on an Illumina HiSeq4000 system by BGI Europe (Copenhagen, Denmark). Read alignment to the human reference genome (GrCH37/hg19) and variant calling was performed using BWA (Li and Durbin 2009) and GATK (Broad Institute, Cambridge, MA, USA) software, respectively. Variant annotation was performed using a custom-designed in-house annotation and variant evaluation pipeline. First, whole exome sequence (WES) data were analyzed for variants in a panel of 142 genes known to be associated with nonsyndromic HI and relatively common syndromic forms of HI (gene list version DG 2.5; Hereditary Hearing Loss Exome Panel Genome Diagnostics Nijmegen-Maastricht, see Online Resource Table S1) in a diagnostic setting. Variants in the 142 deafness-associated genes were classified according to guidelines from the Association for Clinical Genetic Science and the Dutch Society of Clinical Genetic Laboratory Specialists (Wallis et al. 2013). To address other recently published deafness genes, WES data of all subjects were reanalyzed with the most recent version of the gene panel, version DG 2.11, which consists of 159 deafness genes, see Online Resource Table S1. Variants of the complete exome were evaluated according to the criteria described in the supplemental methods section (see Online Resource).

Mean 20x coverage of the enriched regions in WES ranged between 93.5 and 98.0%. Copy number variation (CNV) was addressed by depth of coverage analysis with CoNIFER as described (Krumm et al. 2012; Pfundt et al. 2017). Segregation analysis of selected variants was carried out by Sanger Sequencing, as described (Wesdorp et al. 2018). Primer sequences are available upon request. Confirmation of parental identities in the cases with de novo variants was performed with the AmpFLSTR™ Identifiler™ kit, according to the manufacturer’s protocol (Applied Biosystems, Thermo Fisher Scientific MA, USA).

Sequencing of *CDH23* for individuals I.1 and II.1 of family W17-0883 and III.3 of family W18-0139 was performed by massive parallel ion semiconductor sequencing as described (Diekstra et al. 2015).

## Results

### Loss-of-function variants in ATP2B2 are associated with hearing impairment

**Fig. 1:**
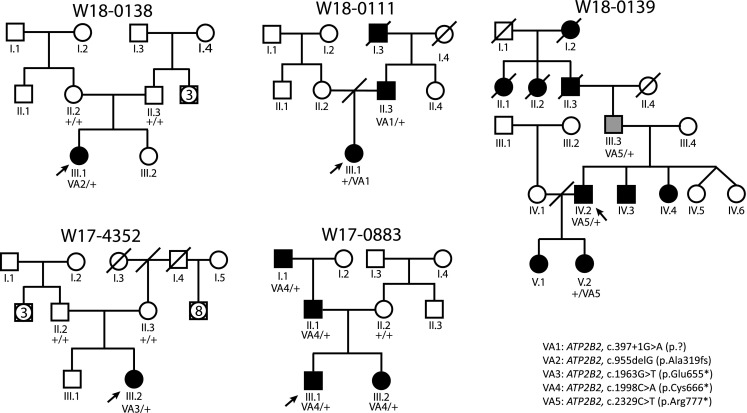
Pedigrees and segregation of *ATP2B2* variants. VA1–VA5, variants in *ATP2B2*, as listed in Table 1 and in this figure. Nomenclature of variants VA1–VA5 is according to transcript NM_001001331.2. The subject marked in grey (III.3, W18-0139) has late-onset hearing impairment, in contrast to all other affected individuals. Subjects IV.3, IV.4 and V.1 of family W18-0139 did not participate in this study. Deceased individuals are considered affected or unaffected by hetero-anamnesis. Index cases are indicated by arrows. +, reference sequence

An otherwise healthy subject of Dutch origin with childhood-onset, rapidly progressive nonsyndromic SNHI (family W18-0138, III.1, Fig. 1) was referred to our out-patient clinic for etiologic consultation. First, *GJB2* was screened in routine diagnostics, which revealed a heterozygous variant of uncertain significance c.647G>T (NM_004004; p.Arg216Ile) [DFNA3A, (MIM 601544); DFNB1, (MIM 220290)]. This variant was not associated with autosomal dominant HI as it was inherited from the unaffected mother. In addition, no variant was identified in the second *GJB2* allele by Sanger sequencing. Therefore, defects in *GJB2* were not considered to underlie the HI in the presented case. Subsequently, WES was performed, which revealed neither other (likely) pathogenic variants in genes of the deafness gene panel (version DG 2.5), nor CNVs of these genes. Finally, analysis of the complete WES data (beyond the gene panel for deafness) was carried out. Truncating variants and variants of canonical splice sites were considered potentially deleterious. Furthermore, other potentially pathogenic variants that met the criteria as provided in the supplemental methods section were considered (See Online Resource). This analysis revealed a truncating variant in *ATP2B2*: c.955delG (NM_001001331.2, p.Ala319fs), which was neither present in our in-house database (~ 20,000 exomes) nor in gnomAD (version r2.02) (Lek et al. 2016). Importantly, the *ATP2B2* variant was not found in the parents and as both parental identities were confirmed, the variant was considered to be de novo (Fig. 1). Several heterozygous missense and truncating defects of the mouse ortholog of *ATP2B2* were associated with early onset, progressive high-frequency HI (Bortolozzi et al. 2010; Carpinelli et al. 2013; Kozel et al. 1998; McCullough and Tempel 2004; Spiden et al. 2008; Street et al. 1998; Takahashi and Kitamura 1999; Tsai et al. 2006; Watson and Tempel 2013; Xu et al. 2011). Therefore, we regarded the *ATP2B2* variant to be an excellent candidate cause of the HI in this subject.

**Table 1:** *ATP2B2* variants identified in this study

Family code	Variant code	Genome	cDNA	Protein	Number of segregations
W18-0111	VA1	Chr3:10452301C>T	c.397+1G>A	p.?	1
W18-0138	VA2	Chr3:10426997delG	c.955delG	p.(Ala319fs)	De novo
W17-4352	VA3	Chr3:10400548C>A	c.1963G>T	p.(Glu655*)	De novo
W17-0883	VA4	Chr3:10400513G>T	c.1998C>A	p.(Cys666*)	3
W18-0139	VA5	Chr3:10391871G>A	c.2329C>T	p.(Arg777*)	2

VA1–VA5, variants in *ATP2B2*, as shown in the pedigrees in Fig. 1. The indicated genomic positions are according to GRCh37/hg19. The cDNA and amino acid positions are based on transcript NM_001001331.2. None of the variants are present in gnomAD version r2.02 or in our in-house WES database of ~ 20.000 exomes

To confirm the association of *ATP2B2* defects with SNHI in humans, WES data of about 700 index cases were analyzed for truncating variants and variants in canonical splice sites in this gene. In 110 of these cases, a dominant inheritance pattern of HI was indicated by the referring clinician. Defects of known genes associated with HI were previously excluded for these cases in routine diagnostics, as indicated in the methods section. Three cases were found to have nonsense variants in *ATP2B2*, and one case a variant in a canonical splice site (Table 1). All four cases had a reported onset of HI in the first decade (Table 2). Segregation analysis of the *ATP2B2* variants in the respective families demonstrated that the c.1963G>T variant in subject III.2 of family W17-4352 was de novo, as it was not detected in the parents (Fig. 1) while both parental identities were confirmed. In families W17-0883 and W18-0111, the identified *ATP2B2* variants co-segregated with the HI in three and one affected family members, respectively. In family W18-0139, the *ATP2B2* variant was present in two participating family members with early onset HI (IV.2 and V2) but also in individual III.3 who reported a late onset of HI, at the age of 55 years.

**Table 2:** Individual results of otoscopy, pure tone audiometry, newborn hearing screening, imaging, and speech discrimination tests

Family	Subject	Newborn screening	Age of onset (y)	Age during study (y)	Otoscopic examination	Imaging		PTA^b^		SRT^b^		Maximum SRS (%)^b^	
						CT	MRI	R	L	R	L	R	L
W18-0111	III.1	Pass	2–5	11	L myringosclerosis		N	22	37	27	47	85	88
	II.3	NT	Cong	45	N			62	62	65	70	92	70
W18-0138	III.1	NT	3	24	N	N	N	105	82	95	57	50	69
W17-4352	III.2	Pass	2	6	N		N	70	75	60	60	70	65
W17-0883	III.1	Pass	4	9	N			33	40	36	40	82	74
	III.2	Pass	6	6	N			17	22	15	19	100	100
	II.1	NT	2–5	32	N	N		62	57	70	72	NT	NT
	I.1^a^	NT	2–5	67	NT			103^a^	NA	NT	NA	NT	NA
W18-0139	V.2	NT	4	17	NT			23	22	10	10	95	100
	IV.2^a^	NT	5	48	N^a^	N	N	60	NA	67	NA	93	NA
	III.3	NT	55	68	N			13	20	13	21	95	95

Age of onset is the reported age of onset in years. Age during study is the age at which subjects had clinical testing for this study

*y* years, *PTA* pure tone average, mean of 0.5, 1 and 2 kHz air conduction thresholds, *R* right, *L* left, *SRT* speech reception threshold, *SRS* speech recognition score, *NT* not tested, *N* normal, *NA* not applicable, *cong*. congenital

^a^Only right ear was assessed

^b^Latest audiogram, made at the age indicated in the fifth column. An exception is individual W18-0138 III.1, for whom the penultimate audiogram at the age of 22 was used because of cochlear implantation in her right ear at the age of 24 years

Other variants that could potentially be causative for HI in the families were addressed (Online Resource Tables S2 and S3). For families W18-0138 and W17-4352, both a dominant and recessive inheritance patterns were addressed. 11 variants met the described criteria and their segregation in the respective families was determined (Online Resources Fig. S1 and Table S2). In families W18-0138 and W17-4352, identified variants with a potentially dominant effect were not de novo but derived from a normal hearing parent and, therefore, considered unlikely to be pathogenic. Variants with a potentially recessive effect were only found in *DNM1* (family W17-4352), a gene that is associated with deafness in mouse (Boumil et al. 2010). However, the variant of one of the alleles was predicted to be synonymous and to have no effect on splicing. In the families with dominantly inherited HI, only one candidate variant, c.3226G>C (p.Asp1076His) in *MYO6*, co-segregated with the HI in family W18-0111.

Since digenic and modifier mechanisms have been described for the combination of *CDH23* and *ATP2B2* variants (Ficarella et al. 2007; Schultz et al. 2005), we evaluated all rare variants in *CDH23* in the index cases. *CDH23* was completely covered in WES by at least ten reads for all coding exons. Six rare variants, named VC1–VC6, were identified and segregation analysis was performed (Online Resource Table S3 and Fig. S1). Four variants (VC1–VC4) are missense variants predicted to have a deleterious effect by at least two of the employed pathogenicity prediction tools. VC2 and VC3 are located in *cis* on the paternal allele and are co-inherited with the *ATP2B2* variant in family W18-0111. VC1 and VC4 co-occurred with the de novo variants in families W18-0138 and W17-4352, respectively. Therefore, no segregation analysis was carried out. For an intronic (VC5) and a synonymous (VC6) variant, no effects on splicing or protein function were predicted. Also, VC5 does not co-segregate with the *ATP2B2* variant (and HI) in family W17-0883. Sequencing of *CDH23* was performed in individuals I.1 and II.1 (W17-0883) to identify other variants in this gene that might contribute the HI. This revealed only common variants. VC6 co-occurred with the *ATP2B2* variant in subjects IV.2 and V2 of family W18-0139, but not in individual III.3. The latter reported to have developed HI around the age of 55 years. Sequencing of *CDH23* in this individual revealed that he carries the VC1 variant heterozygously.

### Characteristics of ATP2B2-associated HI

**Fig. 2:**
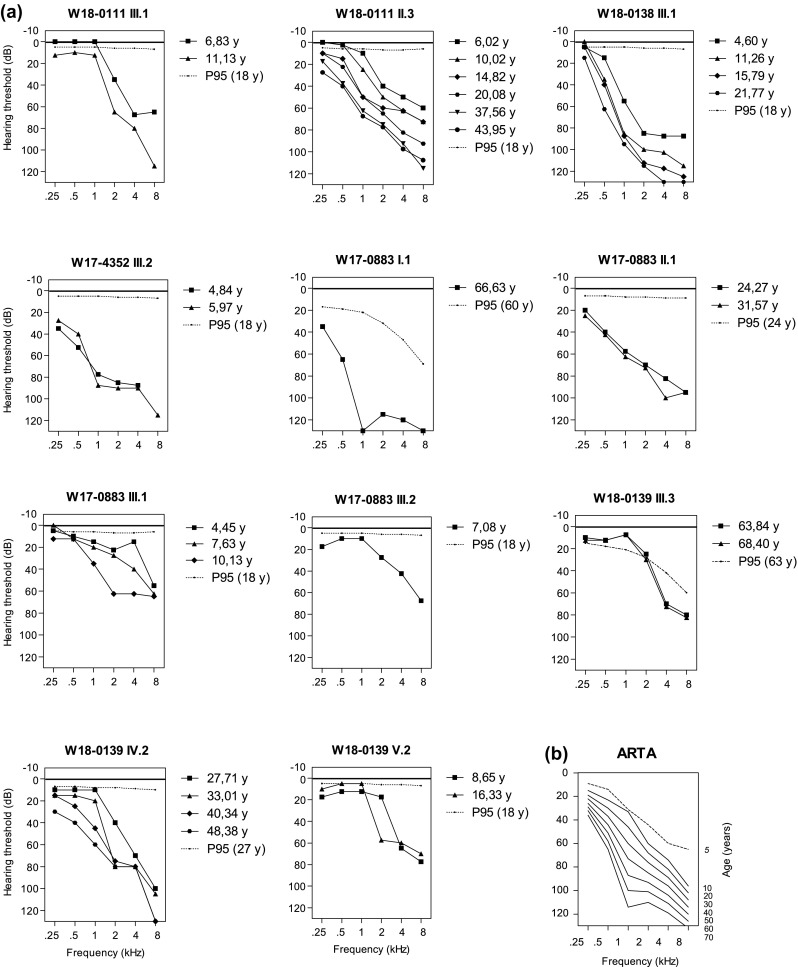
Audiograms of individuals with heterozygous deleterious *ATP2B2* variants. **a** Air conduction thresholds of all participating individuals with a deleterious *ATPB2B2* variant are depicted. For subjects I.1 of family W17-0883 and IV.2 of family W18-0139, only data of their right ears are displayed. For all other subjects, average of left and right ear thresholds is shown. The P95 values are matched to the individuals’ sex and age at first audiometry, according to the ISO 7029:2017 standard. The lowest age for which the ISO 7029:2017 can be applied is 18 years. y, age in years. **b** Age-related typical audiogram (ARTA) representing cross-sectional linear regression analysis of last visit audiograms of all subjects with a deleterious *ATP2B2* variant (*N* = 11). The dashed line represents the average air conduction thresholds at the age of 5

None of the affected subjects or their parents reported exposure to excessive noise, long-term usage of antibiotics, history of head trauma or meningitis. Subjects III.3 and IV.2 of family W18-0139 reported complaints of tinnitus. All four subjects who underwent newborn hearing screening passed the first screening (Table 2). HI in all except two participating affected subjects was bilateral, sensorineural, symmetric, and mild to profound. For individual IV.2 of family W18-139, who repeatedly underwent ear surgery of the left ear, and I.1 of family W17-0883 who had a conductive HI on his left ear due to previous cholesteatoma surgery, only data of the right ears are depicted and included in further evaluations. For the other subjects, mean air conduction threshold values are depicted in Fig. 2 and employed in further analyses. Audiogram configurations were (steeply) downsloping (Fig. 2) and the age of onset was in the first decade with one exception (Table 2). Subject III.3 of family W18-0139 reported an onset age of 55 years, but no audiometry was performed before the age of 64 years. His hearing is clearly below the P95 for presbyacusis for the higher frequencies. Other affected subjects (IV.3, IV.4 and V.1) in this family did not participate in the clinical and genetic evaluations, but on hetero-anamnesis they had an early childhood onset of HI. Subject III.3 reported that his father, paternal aunts and grandmother had HI early in life and that they were wearing hearing aids.

Speech reception thresholds in general were lower than or comparable to pure tone average thresholds (0.5–2 kHz), indicating the absence of retrocochlear pathology. This was confirmed by BERA measurements in four subjects (Online Resource Table S4, data not shown).

An ARTA was calculated to provide insight in the progression of the HI per decade (Fig. 2b). The average annual threshold deterioration (ATD) for the ages 10–70 was 0.5 dB/year for the low frequencies (250–500Hz), 1.1 db/y for middle frequencies (1–2 kHz) and 0.7 dB/year for the high frequencies (4–8 kHz). Under the age of 10 years, audiograms tend to be less reliable due to more prevalent otitis media with effusion or less co-operation or cognitive capabilities of the young subjects. To give insight in the HI at this age, we calculated the average thresholds of all subjects with a hearing test at about 5 years of age (Fig. 2b, dashed line).

Five subjects underwent computed tomography (CT) and/or magnetic resonance imaging (MRI) of the bilateral temporal bones/cochlea and cerebellopontine angle, which revealed normal inner and middle ear anatomy, except for the left ear of subject IV.2 of family W18-0139 for reasons already indicated (Table 2).

### Results of vestibular testing

Vestibular history was obtained from all subjects and extensive vestibular testing of at least one affected subject per family (except for family W17-4352) revealed only minor vestibular abnormalities (see Online Resource Table S4). Two subjects reported vestibular complaints; one was diagnosed with benign paroxysmal position vertigo (BPPV) and the second had complaints due to a perilymph leakage at cholesteatoma surgery. Saccular function could not be measured in subjects III.1 of family W18-0138 (bilaterally), III.1 (left ear) of W18-0111, and IV.2 of W18-0139 (left ear), whereas utricular function was not measurable in III.1 of family W18-0138. Maximum stimulus levels were reached in all these subjects.

## Discussion

In this study, five families were presented, in which four mono-allelic truncating variants and one canonical splice site variant in *ATP2B2* co-segregated with early onset progressive nonsyndromic SNHI and with late-onset SNHI in one subject. All five variants affected the (predicted) ortholog of the w/a isoform of PMCA2, which is the predominant Ca^2+^ATPase in rodent hair cells and specifically localizes to stereocilia (Grati et al. 2006; Hill et al. 2006). In man, transcripts encoding the w/a isoform of PMCA2 have so far not been identified but the w splicing pattern has been shown (ENSEMBL, UCSC genome browser). See Online Resource Fig. S2 for a schematic representation of the different isoforms and positions of the variants. The truncating variants can be predicted to have a loss-of-function effect. This might also be the case for the splice site variant by inducing skipping of exon 3, retention of intron 3 or the use of an alternative exonic or intronic splice site. Skipping of exon 3 (198 nucleotides) is predicted to result in the depletion of 66 amino acids from the N-terminal part of the protein, including the first transmembrane region (See Online Resource Fig. S3 for splice prediction). Two of the truncating variants occurred de novo, which supports pathogenicity of the variants. A causal association with disease is further strengthened by a pLI score of 1.00 reported for *ATP2B2*. This score is an indication of extreme loss of function intolerance of *ATP2B2* (Lek et al. 2016). The DOMINO variant effect prediction tool, predicts *ATP2B2* to be very likely associated with a dominantly inherited disorder (Quinodoz et al. 2017) which is in agreement with the inheritance pattern of HI in the described families.

As digenic inheritance of HI was proposed for mono-allelic missense variants in *ATP2B2* and *CDH23* (Ficarella et al. 2007), we addressed this type of inheritance in the families in our study. Although rare *CDH23* variants co-occurred with *ATP2B2* variants in all five index cases, our findings indicate that mono-allelic loss-of-function variants of *ATP2B2* are the underlying cause of HI. First, the *CDH23* variant VC5 in individuals III.1 and III.2 of family W17-0883 is intronic and predicted not to affect splicing. Furthermore, this variant was not found in the affected family members I.1 and II.1, who also did not carry any other rare *CDH23* variants in the coding sequences or flanking intronic regions. Second, the synonymous *CDH23* variant VC6 in family W18-0139 is predicted not to affect the existing splice sites nor to introduce such sites. Although this variant did co-segregate with early onset HI in the family, subject III.3 with late-onset HI carried a different *CDH23* variant, namely missense variant VC1 that is also seen in the index case of family W18-0138. Third, none of the variants has been classified as (likely) pathogenic in Clinvar (VC1–VC3, (likely) benign; VC4, uncertain significance; VC6, conflicting, UV1/UV2/UV3) and all but one (VC4) occur relatively frequently in gnomAD. Only variants in deep intronic regions or promoter regions were not addressed and can, therefore, not be excluded. CNVs of *CDH2*3 can be excluded for the index cases only. Finally, the affected individual in the family reported by Ficarella et al. was pre-screened only for mutations in *GJB6, MYO6, CDH23*, and the most common mutations in *GJB2* (Ficarella et al. 2007). As many of the known genes associated with recessive HI were not tested, several of which are commonly involved (e.g. *MYO15A*), defects in other deafness genes cannot be excluded in the reported case. We cannot exclude a modifying effect of the *CDH23* variants on HI in the affected subjects in our study, as has been reported for mouse mutants of *Atp2b2* (McCullough and Tempel 2004).

As indicated by the pLI score of 1.00, loss-of-function variants in *ATP2B2* are rare. In gnomAD, eight such variants are reported heterozygously, representing 26 alleles. Three of these variants, representing 21 alleles, affect canonical splice sites of the alternatively spliced exons 7–9 at site A and all three are in frame (see Online Resource Fig. S2). One of these three variants, c.1001-1G>A (NM_001001331.2), was identified in 19 non-Finnish Europeans and is predicted to result in the loss of the splice acceptor site of exon 9. This loss potentially results in skipping of this exon and thereby to a shift from splicing pattern w to y at site A (Online Resource Fig. S2). Alternative splicing at site A determines apical targeting of the encoded PMCA2 in hair cells. When alternative splicing at site A resulted in insertion of at least 31 amino acids, the encoded PMCA2 exclusively targeted to the hair bundle (Grati et al. 2006). As exons 7, 8, and 9 encode 11, 20, and 14 amino acids, respectively, skipping of exon 9 results in 31 amino acids encoded at site A (pattern y) and exclusively apical targeting of the protein (Grati et al. 2006). This suggests that the c.1001-1G>A might have no or a milder phenotypic effect compared to *ATP2B2* variants identified in the present study.

The other two canonical splice site variants in gnomAD, each representing a single allele, are predicted to result in the loss of the splice donor site of exon 7 and the splice acceptor site of exon 8, respectively. Skipping of exon 7 would lead to a PMCA2 protein with 34 amino acids encoded by exons inserted at site A and thus apical targeting according to Grati et al. (Grati et al. 2006). Skipping of exon 8 would result in a PMCA2 protein with both apical and basolateral distribution in hair cells (Grati et al. 2006). In conclusion, 21 of 26 loss-of-function alleles reported in gnomAD might well lead to no or a mild phenotype.

Two de novo truncating variants in *ATP2B2* have been identified in a study on autism spectrum disorders (ASD), which is a complex disorder (Takata et al. 2018). These variants, c.2268C>A (p. Cys756*) and c.3191G>G (p.Trp1064*) according to transcript NM_001001331.2, affect exons 15 and 20 of the transcript, respectively (see Online Resource Fig. S2). Therefore, these variants are predicted to affect all known PMCA2 isoforms and to lead to nonsense-mediated decay. Based on our findings, the de novo *ATP2B2* variants identified in the ASD cases are expected to cause HI but this was not reported by the authors. The age of the subjects was not specified but indicated to be more than 2 years. This suggests that these subjects might not yet have developed HI. Alternatively, if they are toddlers, the HI may have mimicked or contributed to symptoms of ASD due to communication problems as has been reported previously (Worley et al. 2011). The subjects in our study did not display signs of ASD and this condition was neither indicated by themselves nor by their parents.

The type of mutations and their distribution in the gene suggest haploinsufficiency as disease mechanism. 4 of 5 presented *ATP2B2* variants potentially lead to nonsense-mediated decay (NMD). A haploinsufficiency mechanism is further supported by the findings of McCullough et al. (2007) who demonstrated that SNHI in cases with the 3p-syndrome is associated with loss of *ATP2B2* (McCullough et al. 2007). They reported a high-frequency HI with a steeply downsloping audiogram configuration, similar to that in the cases in our study. Malmgren et al. suggested that deletion of *ATP2B2* is not sufficient to cause HI (Malmgren et al. 2007). However, the HI in the cases they reported is not sufficiently characterized to support SNHI in all them. For example, HI of one of the included subjects was indicated to be conductive in the original publication (Angeloni et al. 1999; Malmgren et al. 2007; McCullough et al. 2007).

WES data of index cases were analyzed for other variants in known mouse and/or human deafness genes that could potentially be causative for HI. This revealed only one candidate variant, c.3226G>C (p.Asp1076His) in *MYO6*, that co-segregated with the HI in family W18-0111. Although a phenotypic effect of this variant cannot be excluded, *MYO6* variants with a dominant effect are known to be associated with progressive HI with a different audiogram configuration and in the majority of cases an onset after childhood (Miyagawa et al. 2015; Oonk et al. 2013; Sampaio-Silva et al. 2018; Topsakal et al. 2010). Therefore, the identified variant is unlikely to fully explain the HI in family W18-0111. Also, the HI in family W18-0111 is comparable to that in the other families reported in this study.

13 mouse mutants (*deafwaddler*) have been described for *Atp2b2*, 12 of which have been described to display congenital severe-to-profound HI accompanied by a severe vestibular/ataxic phenotype when the mutation is in the homozygous state. The mutations in all 12 affect the w/a isoform of PMCA2. Mice with loss of function *Atp2b2* mutations in the heterozogous state display a rapidly progressive early onset HI (before the age of 3–5 weeks) with high-frequency hearing being most severely affected (Carpinelli et al. 2013; McCullough and Tempel 2004; Noben-Trauth et al. 1997; Watson and Tempel 2013). The type of HI in these mice is highly similar to that in the presented affected subjects.

Based on the findings in the presented families, it cannot be determined whether hearing of the affected subjects was abnormal at birth, as all four subjects who underwent newborn hearing screening passed the first test. The TEOAE devices employed in the screening are calibrated to detect thresholds of 35 dB HL or more, for the frequencies between 1 and 4 kHz (van der Ploeg et al. 2012). This means that in case the tested newborns with *ATP2B2* defects had elevated thresholds, these were below 35 dB HL for the tested frequencies. Alternatively, only the thresholds for higher frequencies (> 4 kHz) surpassed 35dB, which might have well been the case based on audiogram configuration (Fig. 2b) and the findings in mouse models. Delayed speech development and difficulties at school were the reasons for the parents to have their children’s hearing tested leading to a diagnosis of HI around the age of 2–5 years.

One individual (W18-0139, III.3, Fig. 1) in our study displayed HI with a late onset rather than an early onset. This suggests the existence of modifying (genetic) factors. For mouse, it has been described that small (15%) increases in PMCA2 activity can account for large differences in hearing phenotype (McCullough and Tempel 2004). Therefore, it is tempting to speculate that the late onset of HI in this case might be due to a relatively high inner ear expression of the second *ATP2B2* copy. As subject II.3 was indicated to be severely hearing impaired at the age of 30 years, mosaic presence of the *ATP2B2* variant in subject III.3 seems unlikely to explain the late onset of his HI. However, as HI is genetically highly heterogeneous the deceased family members I.2, II.1, II.2 and II.3 may have had HI with a different genetic aetiology. Identification of additional families with deletions or truncating variants of *ATP2B2* will shed light on the variability of onset age of *ATP2B2*-associated HI.

In hair cells and endolymph, Ca^2+^ is important for maintaining the endocochlear potential, contributes to the mechanotransduction (MET) current, affects MET channel activity, tip-link structure, and, although under debate now, it is thought to play a role in the process of fast adaptation [for review see (Bortolozzi and Mammano 2018; Cali et al. 2017; Qiu and Muller 2018)]. PMCA2 is the Ca^2+^ pump in OHC stereocilia and, therefore, important for Ca^2+^ homeostasis in these structures. As has been demonstrated for several of the *Atp2b2* mutant mice, HI and OHC dysfunctions precede hair cell degeneration and Ca^2+^ cytotoxicity has been hypothesized to underlie the latter (Bortolozzi et al. 2010; Konrad-Martin et al. 2001; Spiden et al. 2008). Degeneration of both OHCs and IHCs was demonstrated to be most severe at the cochlear base in heterozygous as well as homozygous *Atp2b2* mutant mice (Bortolozzi et al. 2010; Spiden et al. 2008). This is in agreement with high-frequency hearing being most severely affected in the presented families. Analogous to what has been described in mice with a heterozygous *Atp2b2* mutation, degeneration of OHCs, IHCs and supporting cells is likely to cause the progression of HI to severe-to-profound in humans.

PMCA2 is also expressed in the stereocilia of vestibular hair cells (Grati et al. 2006; Street et al. 1998). A vestibular phenotype was only seen in homozygous or compound heterozygous and not in heterozygous *Atp2b2* mutant mouse models (Bortolozzi et al. 2010; Carpinelli et al. 2013; Kozel et al. 1998; McCullough and Tempel 2004; Spiden et al. 2008; Street et al. 1998; Sun et al. 2008; Watson and Tempel 2013). This is histologically supported by studies of Takahashi and Kitamura on the *“wri”* mouse model; in *wri*/*wri* homozygotes, severe degeneration is seen in both the cochlea and saccule within 3 months. In heterozygotes, degeneration was only seen in the cochlea (Takahashi and Kitamura 1999). Vestibular complaints were found in only two patients in this study, but can be attributed to other factors than defects in *ATP2B2* (Online Resource Table S4). Oculomotor, vHIT, caloric and rotational chair testing yielded no abnormalities, as expected from previous studies in mouse models. In four subjects, we could not determine whether saccular or utricular function was present. First, we measured until 100dB HL (cVEMP) and 140 dB FL (oVEMP), according to protocol. Higher stimulus levels are too uncomfortable for subjects. The contralateral measurements were close to maximum stimulus levels in subjects III.1 of family W18-0111 and IV.2 of W18-0139. It is, therefore, possible that ipsilateral levels could have been measured with stimuli just above the maximum stimulus levels. Second, in about half of all patients who underwent vestibular examination in our clinic prior to cochlear implantation, it was impossible to measure c- and oVEMPs (Beynon, unpublished data). This relates to subject III.1 of W18-0138, who was the only subject with a cochlear implant. Based on the present findings, we concluded that heterozygous loss-of function variants in *ATP2B2* are unlikely to be associated with vestibular dysfunction.

In conclusion, we presented heterozygous *ATP2B2* defects to be associated with dominantly inherited nonsyndromic SNHI. All but one subject displayed a rapidly progressive high-frequency HI with a congenital or early childhood onset. Four and possibly all five identified variants have a truncating effect. As amino acid substitutions in mouse PMCA2 can lead to loss of function (e.g. Bortolozzi et al. 2010; Spiden et al. 2008), missense variants in *ATP2B2* are to be expected as a cause of HI in humans as well. Variants resulting in reduced PMCA2 function might lead to recessive HI, analogous to the deafwaddler (*dfw*) allele (McCullough and Tempel 2004).

The DOOFNL Consortium consists of M.F. van Dooren, H.H.W. de Gier, E.H. Hoefsloot, M.P. van der Schroeff (ErasmusMC, Rotterdam, The Netherlands), S.G. Kant, L.J.C. Rotteveel (LUMC, Leiden, the Netherlands), J.C.C. Widdershoven, J.R. Hof, E.K. Vanhoutte (MUMC+, Maastricht, The Netherlands), R.J.C. Admiraal, I. Feenstra, H. Kremer, R.J.E. Pennings, H.G. Yntema (Radboudumc, Nijmegen, The Netherlands) R.H. Free and J.S. Klein Wassink-Ruiter (UMCG, Groningen, The Netherlands), R.J. Stokroos, A.L. Smit, M.J. van den Boogaard (UMC, Utrecht, The Netherlands) and F.A. Ebbens, S.M. Maas, A. Plomp, T.P.M. Goderie, P. Merkus and J. van de Kamp (Amsterdam UMC, Amsterdam, The Netherlands).

## Web resources

Alamut Visual, http://www.interactive-biosoftware.com/alamut-visual/. Ensemble Genome browser, https://www.ensembl.org/index.html. GnomAD browser, http://gnomad.broadinstitute.org/. Hereditary Hearing loss Homepage, http://hereditaryhearingloss.org. UCSC Genome browser, https://genome.ucsc.edu/.

## Electronic supplementary material

Below is the link to the electronic supplementary material.

Supplementary material 1 (DOCX 2496 KB)

## References

[CR1] Angeloni D, Lindor NM, Pack S, Latif F, Wei MH, Lerman MI (1999). CALL gene is haploinsufficient in a 3p- syndrome patient. Am J Med Genet.

[CR2] Bortolozzi M, Mammano F (2018). PMCA2 pump mutations and hereditary deafness. Neurosci Lett.

[CR3] Bortolozzi M, Brini M, Parkinson N, Crispino G, Scimemi P, De Siati RD, Di Leva F, Parker A, Ortolano S, Arslan E, Brown SD, Carafoli E, Mammano F (2010). The novel PMCA2 pump mutation Tommy impairs cytosolic calcium clearance in hair cells and links to deafness in mice. J Biol Chem.

[CR4] Bosman AJ, Smoorenburg GF (1995). Intelligibility of Dutch CVC syllables and sentences for listeners with normal hearing and with three types of hearing impairment. Audiology.

[CR5] Boumil RM, Letts VA, Roberts MC, Lenz C, Mahaffey CL, Zhang ZW, Moser T, Frankel WN (2010) A missense mutation in a highly conserved alternate exon of dynamin-1 causes epilepsy in fitful mice. PLoS Genet 6. 10.1371/journal.pgen.1001046 10.1371/journal.pgen.1001046 PMC2916854 20700442

[CR6] Brown SD, Hardisty-Hughes RE, Mburu P (2008). Quiet as a mouse: dissecting the molecular and genetic basis of hearing. Nat Rev Genet.

[CR7] Cali T, Brini M, Carafoli E (2017). The PMCA pumps in genetically determined neuronal pathologies. Neurosci Lett.

[CR8] Carpinelli MR, Manning MG, Kile BT, Burt RA (2013). Two ENU-induced alleles of Atp2b2 cause deafness in mice. PLoS One.

[CR9] Diekstra A, Bosgoed E, Rikken A, van Lier B, Kamsteeg EJ, Tychon M, Derks RC, van Soest RA, Mensenkamp AR, Scheffer H, Neveling K, Nelen MR (2015). Translating sanger-based routine DNA diagnostics into generic massive parallel ion semiconductor sequencing. Clin Chem.

[CR10] Ficarella R, Di Leva F, Bortolozzi M, Ortolano S, Donaudy F, Petrillo M, Melchionda S, Lelli A, Domi T, Fedrizzi L, Lim D, Shull GE, Gasparini P, Brini M, Mammano F, Carafoli E (2007). A functional study of plasma-membrane calcium-pump isoform 2 mutants causing digenic deafness. Proc Natl Acad Sci USA.

[CR11] Friedman LM, Dror AA, Avraham KB (2007). Mouse models to study inner ear development and hereditary hearing loss. Int J Dev Biol.

[CR12] Grati M, Aggarwal N, Strehler EE, Wenthold RJ (2006). Molecular determinants for differential membrane trafficking of PMCA1 and PMCA2 in mammalian hair cells. J Cell Sci.

[CR13] Hill JK, Williams DE, LeMasurier M, Dumont RA, Strehler EE, Gillespie PG (2006). Splice-site A choice targets plasma-membrane Ca2+-ATPase isoform 2 to hair bundles. J Neurosci.

[CR14] Huygen PLM, Pennings RJE, Cremers CW (2003). Characterizing and distinguishing progressive phenotypes in nonsyndromic autosomal dominant hearing impairment. Audiol Med.

[CR15] Konrad-Martin D, Norton SJ, Mascher KE, Tempel BL (2001). Effects of PMCA2 mutation on DPOAE amplitudes and latencies in deafwaddler mice. Hear Res.

[CR16] Kozel P, Friedman R, Erway L, Yamoah E, Liu L, Riddle T, Duffy J, Doetschman T, Miller M, Cardell E, Shull G (1998). Balance and hearing deficits in mice with a null mutation in the gene encoding plasma membrane Ca^2+^-ATPase isoform 2. J Biol Chem.

[CR17] Krumm N, Sudmant PH, Ko A, O’, Roak BJ, Malig M, Coe BP, Project NES, Quinlan AR, Nickerson DA, Eichler EE (2012). Copy number variation detection and genotyping from exome sequence data. Genome Res.

[CR18] Lek M, Karczewski KJ, Minikel EV, Samocha KE, Banks E, Fennell T, O’Donnell-Luria AH, Ware JS, Hill AJ, Cummings BB, Tukiainen T, Birnbaum DP, Kosmicki JA, Duncan LE, Estrada K, Zhao F, Zou J, Pierce-Hoffman E, Berghout J, Cooper DN, Deflaux N, DePristo M, Do R, Flannick J, Fromer M, Gauthier L, Goldstein J, Gupta N, Howrigan D, Kiezun A, Kurki MI, Moonshine AL, Natarajan P, Orozco L, Peloso GM, Poplin R, Rivas MA, Ruano-Rubio V, Rose SA, Ruderfer DM, Shakir K, Stenson PD, Stevens C, Thomas BP, Tiao G, Tusie-Luna MT, Weisburd B, Won HH, Yu D, Altshuler DM, Ardissino D, Boehnke M, Danesh J, Donnelly S, Elosua R, Florez JC, Gabriel SB, Getz G, Glatt SJ, Hultman CM, Kathiresan S, Laakso M, McCarroll S, McCarthy MI, McGovern D, McPherson R, Neale BM, Palotie A, Purcell SM, Saleheen D, Scharf JM, Sklar P, Sullivan PF, Tuomilehto J, Tsuang MT, Watkins HC, Wilson JG, Daly MJ, MacArthur DG, Exome Aggregation C (2016). Analysis of protein-coding genetic variation in 60,706 humans. Nature.

[CR19] Li H, Durbin R (2009). Fast and accurate short read alignment with Burrows-Wheeler transform. Bioinformatics.

[CR20] Malmgren H, Sahlen S, Wide K, Lundvall M, Blennow E (2007). Distal 3p deletion syndrome: detailed molecular cytogenetic and clinical characterization of three small distal deletions and review. Am J Med Genet A.

[CR21] Mazzoli M, Van Camp G, Newton V, Giarbini N, Declau F, Parving A (2003). Recommendations for the description of genetic and audiological data for families with nonsyndromic hereditary hearing impairment. Audiol Med.

[CR22] McCullough BJ, Tempel BL (2004). Haplo-insufficiency revealed in deafwaddler mice when tested for hearing loss and ataxia. Hear Res.

[CR23] McCullough BJ, Adams JC, Shilling DJ, Feeney MP, Sie KC, Tempel BL (2007). 3p-syndrome defines a hearing loss locus in 3p25.3. Hear Res.

[CR24] Miyagawa M, Nishio SY, Kumakawa K, Usami S (2015). Massively parallel DNA sequencing successfully identified seven families with deafness-associated MYO6 mutations: the mutational spectrum and clinical characteristics. Ann Otol Rhinol Laryngol.

[CR25] Noben-Trauth K, Zheng QY, Johnson KR, Nishina PM (1997). mdfw: a deafness susceptibility locus that interacts with deaf waddler (dfw). Genomics.

[CR26] Oonk AM, Leijendeckers JM, Lammers EM, Weegerink NJ, Oostrik J, Beynon AJ, Huygen PL, Kunst HP, Kremer H, Snik AF, Pennings RJ (2013). Progressive hereditary hearing impairment caused by a MYO6 mutation resembles presbyacusis. Hear Res.

[CR27] Oonk AM, Beynon AJ, Peters TA, Kunst HP, Admiraal RJ, Kremer H, Verbist B, Pennings RJ (2015). Vestibular function and temporal bone imaging in DFNB1. Hear Res.

[CR28] Papathanasiou ES, Murofushi T, Akin FW, Colebatch JG (2014). International guidelines for the clinical application of cervical vestibular evoked myogenic potentials: an expert consensus report. Clin Neurophysiol.

[CR29] Pfundt R, Del Rosario M, Vissers L, Kwint MP, Janssen IM, de Leeuw N, Yntema HG, Nelen MR, Lugtenberg D, Kamsteeg EJ, Wieskamp N, Stegmann APA, Stevens SJC, Rodenburg RJT, Simons A, Mensenkamp AR, Rinne T, Gilissen C, Scheffer H, Veltman JAPD, Hehir-Kwa JY (2017). Detection of clinically relevant copy-number variants by exome sequencing in a large cohort of genetic disorders. Genet Med.

[CR30] Qiu X, Muller U (2018). Mechanically gated ion channels in mammalian hair cells. Front Cell Neurosci.

[CR31] Quinodoz M, Royer-Bertrand B, Cisarova K, Di Gioia SA, Superti-Furga A, Rivolta C (2017). DOMINO: using machine learning to predict genes associated with dominant disorders. Am J Hum Genet.

[CR33] Sampaio-Silva J, Batissoco AC, Jesus-Santos R, Abath-Neto O, Scarpelli LC, Nishimura PY, Galindo LT, Bento RF, Oiticica J, Lezirovitz K (2018). Exome sequencing identifies a novel nonsense mutation of MYO6 as the cause of deafness in a Brazilian family. Ann Hum Genet.

[CR34] Schultz JM, Yang Y, Caride AJ, Filoteo AG, Penheiter AR, Lagziel A, Morell RJ, Mohiddin SA, Fananapazir L, Madeo AC, Penniston JT, Griffith AJ (2005). Modification of human hearing loss by plasma-membrane calcium pump PMCA2. N Engl J Med.

[CR35] Smith RJ, Bale JF, White KR (2005). Sensorineural hearing loss in children. Lancet.

[CR36] Spiden SL, Bortolozzi M, Di Leva F, de Angelis MH, Fuchs H, Lim D, Ortolano S, Ingham NJ, Brini M, Carafoli E, Mammano F, Steel KP (2008). The novel mouse mutation Oblivion inactivates the PMCA2 pump and causes progressive hearing loss. PLoS Genet.

[CR37] Street VA, McKee-Johnson JW, Fonseca RC, Tempel BL, Noben-Trauth K (1998). Mutations in a plasma membrane Ca2+-ATPase gene cause deafness in deafwaddler mice. Nat Genet.

[CR38] Strehler EE, Zacharias DA (2001). Role of alternative splicing in generating isoform diversity among plasma membrane calcium pumps. Physiol Rev.

[CR39] Sun XY, Chen ZY, Hayashi Y, Kanou Y, Takagishi Y, Oda S, Murata Y (2008). Insertion of an intracisternal A particle retrotransposon element in plasma membrane calcium ATPase 2 gene attenuates its expression and produces an ataxic phenotype in joggle mutant mice. Gene.

[CR40] Takahashi K, Kitamura K (1999). A point mutation in a plasma membrane Ca(2+)-ATPase gene causes deafness in Wriggle Mouse Sagami. Biochem Biophys Res Commun.

[CR41] Takata A, Miyake N, Tsurusaki Y, Fukai R, Miyatake S, Koshimizu E, Kushima I, Okada T, Morikawa M, Uno Y, Ishizuka K, Nakamura K, Tsujii M, Yoshikawa T, Toyota T, Okamoto N, Hiraki Y, Hashimoto R, Yasuda Y, Saitoh S, Ohashi K, Sakai Y, Ohga S, Hara T, Kato M, Nakamura K, Ito A, Seiwa C, Shirahata E, Osaka H, Matsumoto A, Takeshita S, Tohyama J, Saikusa T, Matsuishi T, Nakamura T, Tsuboi T, Kato T, Suzuki T, Saitsu H, Nakashima M, Mizuguchi T, Tanaka F, Mori N, Ozaki N, Matsumoto N (2018). Integrative analyses of de novo mutations provide deeper biological insights into autism spectrum disorder. Cell Rep.

[CR42] Topsakal V, Hilgert N, van Dinther J, Tranebjaerg L, Rendtorff ND, Zarowski A, Offeciers E, Van Camp G, van de Heyning P (2010). Genotype-phenotype correlation for DFNA22: characterization of non-syndromic, autosomal dominant, progressive sensorineural hearing loss due to MYO6 mutations. Audiol Neurootol.

[CR43] Tsai YS, Pendse A, Moy SS, Mohri I, Perez A, Crawley JN, Suzuki K, Maeda N (2006). A de novo deafwaddler mutation of Pmca2 arising in ES cells and hitchhiking with a targeted modification of the Pparg gene. Mamm Genome.

[CR44] van der Ploeg CP, Uilenburg NN, Kauffman-de Boer MA, Oudesluys-Murphy AM, Verkerk PH (2012). Newborn hearing screening in youth health care in The Netherlands: National results of implementation and follow-up. Int J Audiol.

[CR45] Vanspauwen R, Wuyts FL, Krijger S, Maes LK (2017). Comparison of different electrode configurations for the oVEMP with bone-conducted vibration. Ear Hear.

[CR46] Vicario M, Zanni G, Vallese F, Santorelli F, Grinzato A, Cieri D, Berto P, Frizzarin M, Lopreiato R, Zonta F, Ferro S, Sandre M, Marin O, Ruzzene M, Bertini E, Zanotti G, Brini M, Cali T, Carafoli E (2018). A V1143F mutation in the neuronal-enriched isoform 2 of the PMCA pump is linked with ataxia. Neurobiol Dis.

[CR47] Wallis Y, Payne S, McAnulty C, Bodmer D, Sistermans E, Robertson K, Moore D, Abbs S, Deans Z, Devereau A (2013) Practice guidelines for the evaluation of pathogenicity and the reporting of sequence variants in clinical molecular genetics. ACGS/VGKL. Available at http://www.acgs.uk.com/media/774853/evaluation_and_reporting_of_sequence_variants_bpgs_june_2013_-_finalpdf.pdf. Accessed 1 Apr 2018

[CR48] Watson CJ, Tempel BL (2013). A new Atp2b2 deafwaddler allele, dfw(i5), interacts strongly with Cdh23 and other auditory modifiers. Hear Res.

[CR49] Wesdorp M, Murillo-Cuesta S, Peters T, Celaya AM, Oonk A, Schraders M, Oostrik J, Gomez-Rosas E, Beynon AJ, Hartel BP, Okkersen K, Koenen H, Weeda J, Lelieveld S, Voermans NC, Joosten I, Hoyng CB, Lichtner P, Kunst HPM, Feenstra I, de Bruijn SE, Consortium D, Admiraal RJC, Yntema HG, van Wijk E, Del Castillo I, Serra P, Varela-Nieto I, Pennings RJE, Kremer H (2018). MPZL2, encoding the epithelial junctional protein myelin protein Zero-like 2, Is essential for hearing in man and mouse. Am J Hum Genet.

[CR50] WHO (2008). The global burden of disease: 2004 update.

[CR51] Worley JA, Matson JL, Kozlowski AM (2011). The effects of hearing impairment on symptoms of autism in toddlers. Dev Neurorehabil.

[CR52] Wroblewska-Seniuk KE, Dabrowski P, Szyfter W, Mazela J (2017). Universal newborn hearing screening: methods and results, obstacles, and benefits. Pediatr Res.

[CR53] Xu L, Wang Z, Xiong X, Gu X, Gao X, Gao X (2011). Identification of a novel point mutation of mouse Atp2b2 induced by N-ethyl-N-nitrosourea mutagenesis. Exp Anim.

[CR54] Zazo Seco C, Wesdorp M, Feenstra I, Pfundt R, Hehir-Kwa JY, Lelieveld SH, Castelein S, Gilissen C, de Wijs IJ, Admiraal RJ, Pennings RJ, Kunst HP, van de Kamp JM, Tamminga S, Houweling AC, Plomp AS, Maas SM, de Koning Gans PA, Kant SG, de Geus CM, Frints SG, Vanhoutte EK, van Dooren MF, van den Boogaard MH, Scheffer H, Nelen M, Kremer H, Hoefsloot L, Schraders M, Yntema HG (2017). The diagnostic yield of whole-exome sequencing targeting a gene panel for hearing impairment in The Netherlands. Eur J Hum Genet.

