# Supplementary material for: De novo and inherited loss-of-function variants of ATP2B2 are associated with rapidly progressive hearing impairment
Source: Hum Genet. 2018 Dec 8;138(1):61–72. doi: 10.1007/s00439-018-1965-1 (PMC6514080; doi:10.1007/s00439-018-1965-1)
Supplement: Supplementary file 1 — Supplementary material 1 (DOCX 2496 KB) [file 439_2018_1965_MOESM1_ESM.docx]

**Online Resource**

***De novo* and inherited loss-of-function variants of *ATP2B2* are associated with rapidly progressive hearing impairment**

*Human Genetics*

Jeroen J. Smits^1,2^, Jaap Oostrik^1,2^, Andy J. Beynon^1^, Sarina G. Kant^3^, Pia A.M. de Koning Gans^3^, Liselotte J.C. Rotteveel^4^, Jolien S. Klein Wassink-Ruiter^5^, Rolien H. Free^6^ Saskia M. Maas^7^, Jiddeke van de Kamp^7^, Paul Merkus^8^, DOOFNL Consortium, Wouter Koole^9^, Ilse Feenstra^9^, Ronald J.C. Admiraal^1^, Cornelis P. Lanting^1^, Margit Schraders^1,9^, Helger G. Yntema^2,9^, Ronald J.E. Pennings^1,2^*, Hannie Kremer^1,2,9^*

^1^Hearing & Genes, Department of Otorhinolaryngology, Radboud University Medical Center, Nijmegen, the Netherlands; ^2^Donders Institute for Brain, Cognition and Behaviour, Radboud University Medical Center, Nijmegen, The Netherlands; ^3^Department of Clinical Genetics, Leiden University Medical Center, Leiden, the Netherlands; ^4^ Department of Otolaryngology, Head and Neck Surgery, LUMC, Leiden, The Netherlands; ^5^Department of Clinical Genetics, University Medical Center Groningen, Groningen, the Netherlands; ^6^Department of Otolaryngology, Head and Neck Surgery, University Medical Center Groningen, Groningen, the Netherlands ^7^Amsterdam UMC, Vrije Universiteit Amsterdam, Department of Clinical Genetics, Amsterdam, the Netherlands, ^8^Amsterdam UMC, Vrije Universiteit Amsterdam, Otolaryngology – Head and Neck surgery, Ear & Hearing, Amsterdam Public Health research institute, Amsterdam, the Netherlands, ^9^Department of Human Genetics, Radboud University Medical Center, Nijmegen, The Netherlands;

*These authors contributed equally to this work

Corresponding author

Prof. dr. H. Kremer

[Hannie.Kremer@radboudumc.nl](mailto:Hannie.Kremer@radboudumc.nl)

Radboudumc, Nijmegen, The Netherlands.

**Supplemental Methods**

The following criteria have been applied to select candidate variants with a dominant effect detected in WES: allele frequency ≤0.05% in GnomAD (version r2.02) and in the in-house database (~20,000 exomes), ≥ 5 variant reads, % variant reads ≥ 20 and ≤ 90, located in exonic regions and canonical splice sites of genes known to be associated with hearing loss in humans and/or mice. Synonymous variants were excluded, except those in *CDH23*.

For families W17-4352 and W18-0138, also candidate variants compliant with recessive inheritance have been selected as follows: ≥2 variants in a gene, allele frequency ≤1% in GnomAD (version r2.02) and in the in-house database (~20,000 exomes), ≥ 5 variant reads, located in exonic regions and canonical splice sites of genes known to be associated with hearing loss in humans and/or mice considered. For homozygous variants % variant reads had to be ≥ 80 and for compound heterozygous variants ≥ 20 and ≤ 90. Synonymous variants were excluded, except those in *CDH23*.

Prediction of a potential pathogenic effects of missense variants was performed with CADD PHRED (≥15), SIFT (≤0.05), PolyPhen-2 (PPH2, ≥0.450) and Mutation Taster (deleterious). Values for predicted pathogenicity are indicated between brackets. Segregation analysis was performed if at least two of the tools predicted a pathogenic effect of the variant. In the evaluation of candidate variants for recessive inheritance, segregation analysis was performed if the pathogenicity criteria were met for one of the variants. Segregation analysis was performed for all rare *CDH23* variants. A potential effect on splicing was predicted with the tools SpliceSiteFinder-like, MaxEntScan, NNSPLICE, GeneSplicer, and Human Splicing Finder as available in Alamut Visual (version 2.10, Interactive Biosoftware, Rouen, France). A change of at least 30% of splice site scores in at least two of the tools was regarded significant. Also PPH2 and SIFT were employed via Alamut Visual.

**
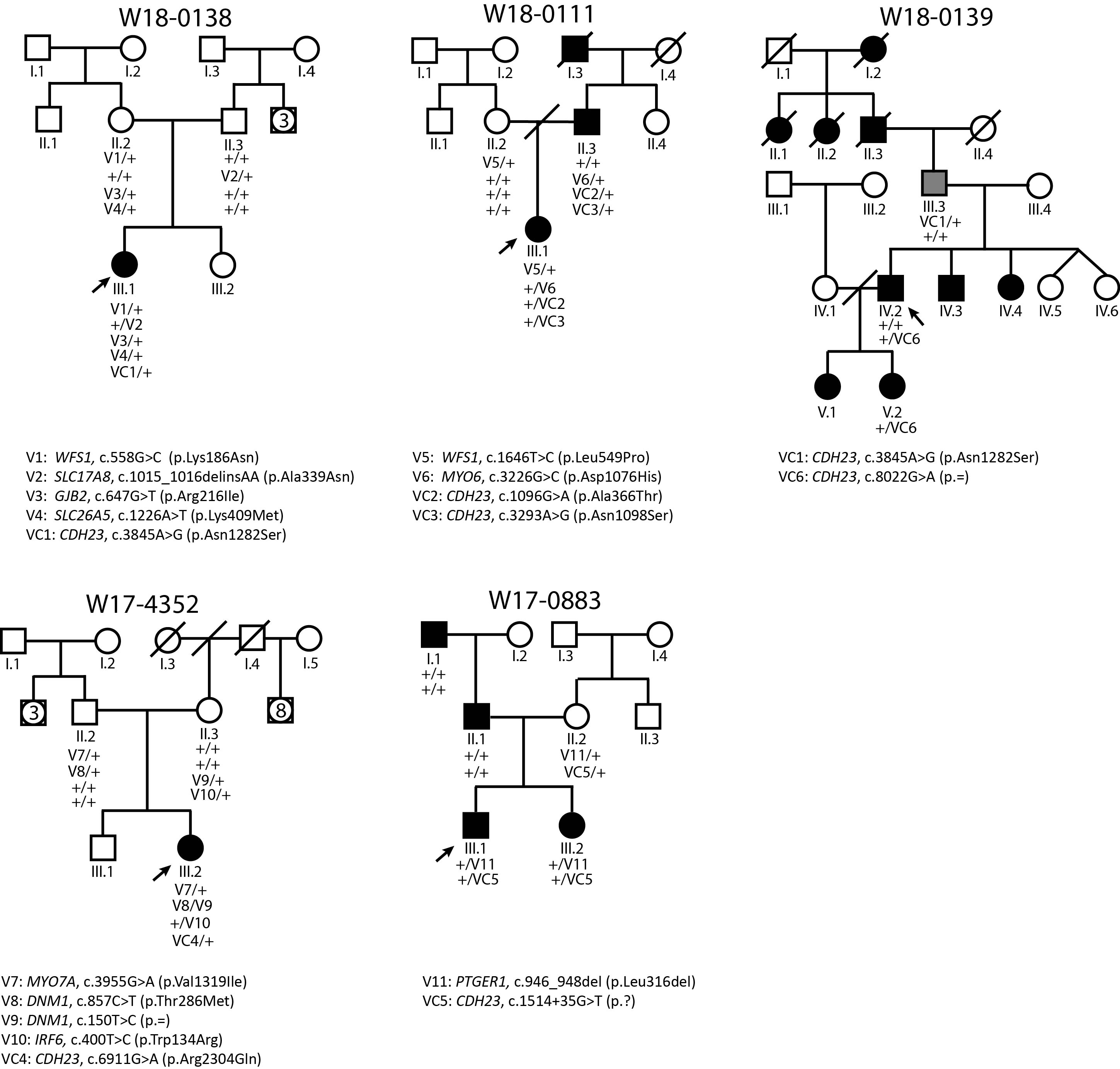
**

**Fig. S1 Segregation of variants identified in WES**

Segregation analysis of selected variants identified in WES. The subject marked in grey (III.3, W18-0139) has late onset hearing impairment, in contrast to the early onset of hearing impairment in all other affected individuals. Affected individuals IV.3, IV.4 and V.1 of family W18-0139 did not participate in this study. Deceased individuals are considered affected or unaffected by heteroanamnesis. Index cases are indicated by arrows. Full details of the variants are provided in Tables S2 and S3. +, reference sequence. V, variant; VC, variant in *CDH23*.


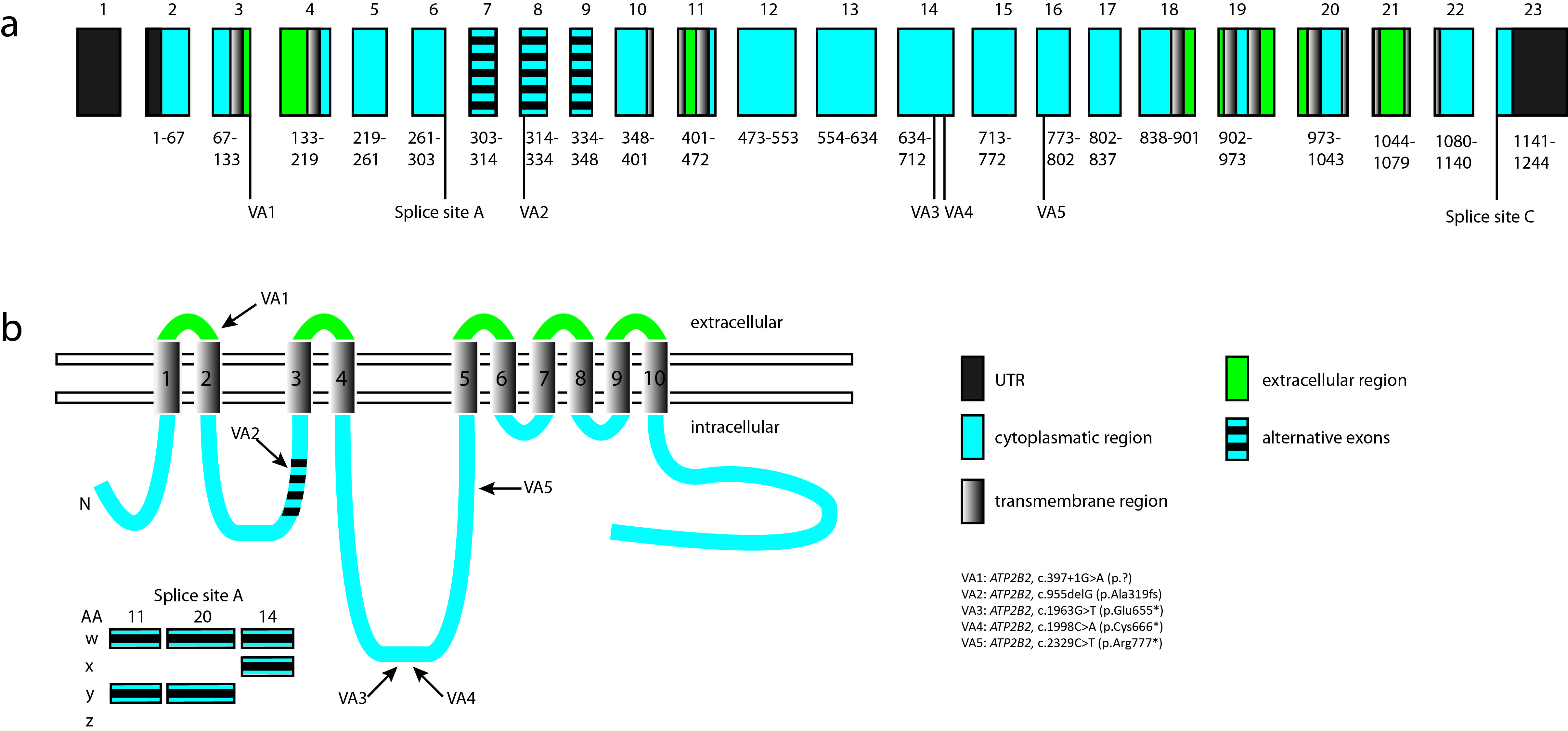


**Fig. S2 Exonic and protein structure of *ATP2B2/*PMCA2**

**(a)** Schematic representation of the genomic structure of *ATP2B2* transcript NM_001001331.3. Exons are numbered and alternatively spliced exons are striped. Numbers 1 to 1243 represent the encoded amino acid residues. Colours represent the encoded protein domains as shown in b, domains were extracted from Uniprot. **(b)** Schematic representation of PMCA2 as encoded by transcript NM_001001331.3. This transcript contains alternatively spliced exons 7-9 at splice site A (w isoform), no alternatively spliced exons at splice site C. In man, the transcript orthologous to the *w/a* splice pattern in mouse has not been identified (UCSC and ENSEMBL Genome browsers). Splice variants at site A (w, x, y, z) are according to those described for mouse (Strehler and Zacharias 2001). Variants identified in this study are indicated as VA1-VA5 (see also Table 1). AA, amino acids; C, C terminus; N, N terminus; UTR, untranslated region; VA, variant in *ATP2B2*.


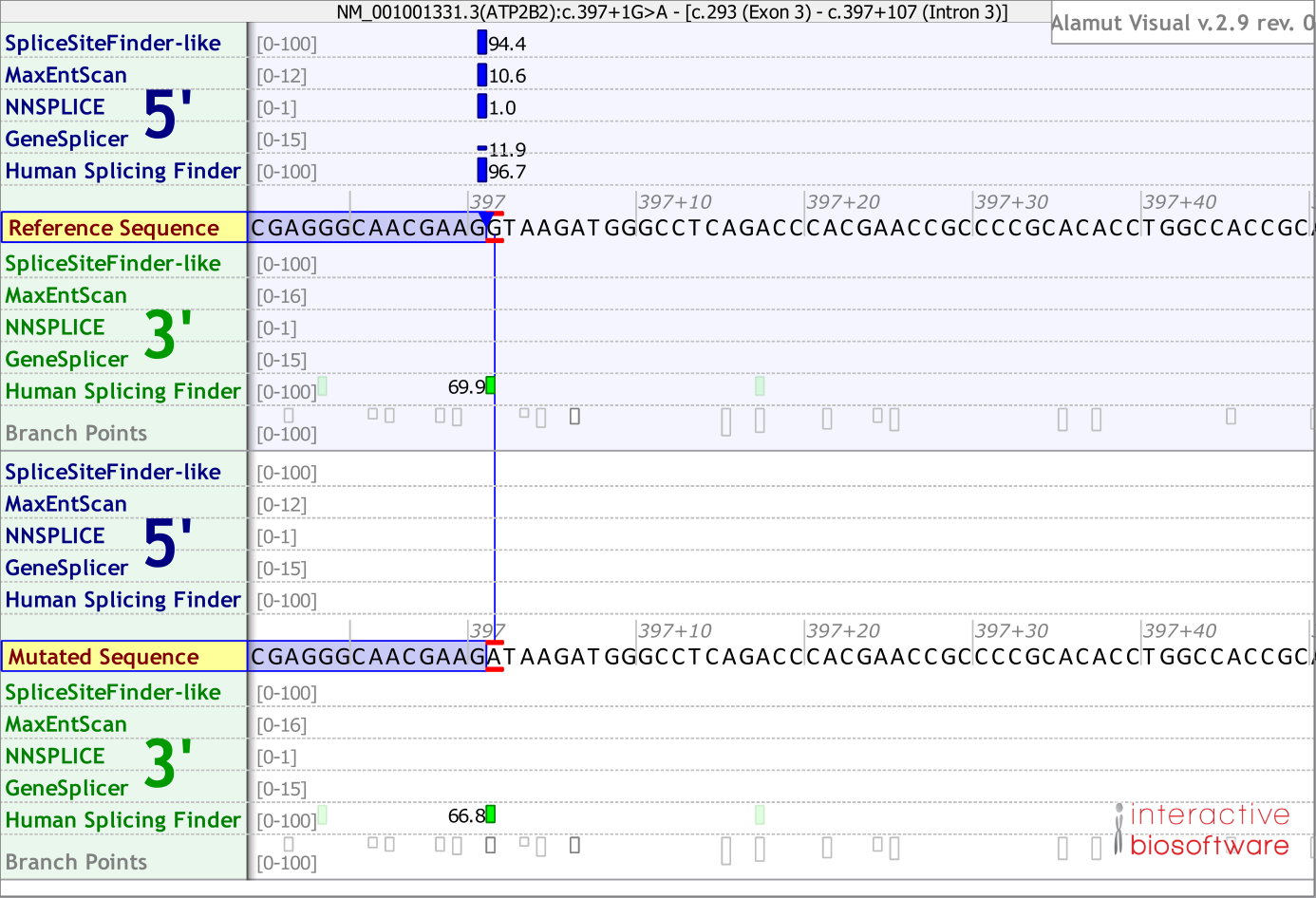


**Fig. S3 Splice prediction**

The variant c.397+1G>A (p.(?)) is predicted to cause loss of the splice donor site of exon 3. Splice site prediction scores and figure were obtained from AlamutVisual version 2.10 (Interactive Biosoftware, Rouen, France).

**Table S1. Hearing impairment gene panel DG 2.11**

| **Gene name** | **Gene name** | **Gene name** | **Gene name** | **Gene name** |
| --- | --- | --- | --- | --- |
|  | *(continued*) | *(continued*) | *(continued*) | *(continued*) |
| *ACTB* | *DIAPH1* | *KITLG* | *PRPS1* | *WBP2* |
| *ACTG1* | *DIAPH3* | *LARS2* | *PTPRQ* | *WFS1* |
| *ADCY1* | *DMXL2^a^* | *LHFPL5* | *RAI1^a^* | *YAP1* |
| *AIFM1* | *DSPP* | *LMX1A^a^* | *RDX* |  |
| *APOPT1* | *EDN3* | *LOXHD1* | *ROR1^a^* |  |
| *ATP2B2^a^* | *EDNRB* | *LRP5^a^* | *S1PR2* |  |
| *ATP6V1B1* | *ELMOD3* | *LRTOMT* | *SERPINB6* |  |
| *BCS1L^a^* | *EPS8* | *MARVELD2* | *SIX1* |  |
| *BDP1* | *EPS8L2^a^* | *MCM2* | *SIX5* |  |
| *BSND* | *ESPN* | *MET^a^* | *SLC17A8* |  |
| *CABP2* | *ESRP1^a^* | *MIR96* | *SLC22A4^a^* |  |
| *CACNA1D* | *ESRRB* | *MITF* | *SLC26A4* |  |
| *CCDC50* | *EYA1* | *MPZL2^a^* | *SLC26A5* |  |
| *CD164* | *EYA4* | *MSRB3* | *SLC29A3^a^* |  |
| *CDC14A^a^* | *FAM65B* | *MYH14* | *SLC33A1* |  |
| *CDH23* | *FGF3* | *MYH9* | *SLC44A4^a^* |  |
| *CEACAM16* | *FOXI1* | *MYO15A* | *SLITRK6* |  |
| *CEP78^a^* | *GAB1^a^* | *MYO3A* | *SMPX* |  |
| *CIB2* | *GATA3^a^* | *MYO6* | *SNAI2* |  |
| *CLDN14* | *GIPC3* | *MYO7A* | *SOX10* |  |
| *CLIC5* | *GJB2* | *NARS2* | *STRC* |  |
| *CLPP* | *GJB3* | *NLRP3* | *SYNE4* |  |
| *CLRN1* | *GJB6* | *OPA1* | *TBC1D24* |  |
| *COCH* | *GPR98* | *OSBPL2* | *TECTA* |  |
| *COL11A1* | *GPSM2* | *OTOA* | *TIMM8A* |  |
| *COL11A2* | *GRHL2* | *OTOF* | *TJP2* |  |
| *COL2A1* | *GRXCR1* | *OTOG* | *TMC1* |  |
| *COL4A3* | *GRXCR2* | *OTOGL* | *TMEM132E* |  |
| *COL4A4* | *HARS* | *P2RX2* | *TMIE* |  |
| *COL4A5* | *HARS2* | *PAX3* | *TMPRSS3* |  |
| *COL4A6* | *HGF* | *PCDH15* | *TMTC2^a^* |  |
| *COL9A1* | *HOMER2* | *PDZD7* | *TNC* |  |
| *COL9A2* | *HSD17B4* | *PET100* | *TPRN* |  |
| *CRYM* | *ILDR1* | *PEX1^a^* | *TRIOBP* |  |
| *DCDC2* | *KARS* | *PEX6^a^* | *TSPEAR* |  |
| *DFNA5* | *KCNE1* | *PNPT1* | *TYR* |  |
| *DFNB31* | *KCNJ10* | *POU3F4* | *USH1C* |  |
| *DFNB59* | *KCNQ1* | *POU4F3* | *USH1G* |  |
| *DIABLO* | *KCNQ4* | *PRKCB^a^* | *USH2A* |  |

Hereditary hearing impairment gene panel version DG-2.11, as used by Genome Diagnostics Nijmegen-Maastricht. The panel consists of 159 genes, both syndromic and non-syndromic forms of hereditary hearing impairment. Underlined genes are associated with HI in an autosomal dominant inheritance pattern. ^a^Genes that have been added to the list since gene panel version DG-2.5, including *ATP2B2*.

**Table S2. Rare variants in known human and mouse deafness genes in the index cases**

| **Family code** | **Variant code^a^** | **Gene name** | **transcript** | **Genome** | **cDNA** | **Protein** | **CADD_PHREDD^b^** | **SIFT^c^** | **PPH2^d^** | **Mutation Taster^e^** | **In house frequency (%)** | **gnomAD-E EAS (%)** | **gnomAD** **-E NFE (%)** |
| --- | --- | --- | --- | --- | --- | --- | --- | --- | --- | --- | --- | --- | --- |
| W18-0138 | V1 | *WFS1* | NM_006005.3 | Chr4: 6293021G>C | c.558G>C | p.Lys186Asn | **18.4** | 0.48 | **0.725** | **Disease causing** | 0.012 | NA | 0.002 |
|  | V2 | *SLC17A8* | NM_139319.2 | Chr12: 100796485_100796486delinsAA | c.1015_1016delinsAA | p.Ala339Asn | **33** | **0.04** | **0.940** | NA | 0.049 | NA | 0.029 |
|  | V3 | *GJB2* | NM_004004.5 | Chr13: 20763074C>A | c.647G>T | p.Arg216Ile | 13.4 | **0.01** | 0.155 | **Disease causing** | 0.037 | NA | 0.007 |
|  | V4 | *SLC26A5* | NM_198999.2 | Chr7: 103032076T>C | c.1226A>T | p.Lys409Met | **24** | 0.09 | **0.997** | **Disease causing** | 0.004 | NA | 0.005 |
| W18-0111 | V5 | *WFS1* | NM_006005.3 | Chr4: 6303168T>C | c.1646T>C | p.Leu549Pro | 11.97 | 0.13 | 0.331 | **Disease causing** | 0.008 | NA | 0.003 |
|  | V6 | *MYO6* | NM_004999.3 | Chr6:76617371G>C | c.3226G>C | p.Asp1076His | **20.4** | **0.00** | **0.997** | **Disease causing** | 0.008 | NA | 0.001 |
| W17-4352^f^ | V7 | *MYO7A* | NM_000260.3 | Chr11:76903126G>A | c.3955G>A | p.Val1319Ile | **31** | 0.25 | **0.926** | **Disease causing** | 0.004 | 0.000 | 0.002 |
|  | V8 | *DNM1* | NM_004408.3 | Chr9 :130984483C>T | c.857C>T | p.Thr286Met | **21** | 0.08 | **1.000** | Polymor-phism | 0.004 | 0.000 | 0.000 |
|  | V9 | *DNM1* | NM_004408.3 | Chr9:130965899T>C | c.150T>C | p.= | 9 | NA | NA | NA | 0.004 | 0.000 | - |
|  | V10 | *IRF6* | NM_006147.3 | Chr1:209968743A>G | c.400T>C | p.Trp134Arg | **15.4** | **0.01** | NA | **Disease causing** | 0.004 | 0.000 | 0.000 |
| W17-0883 | V11 | *PTGER1* | NM_000955.2 | Chr19:14583633_14583635del | c.946_948del | p.Leu316del | **36** | NA | NA | NA | 0 | NA | 0.002 |

In family W17-4352 GnomAD East Asian was also assessed due to ancestry. Genomic positions are according to GRCh37/hg19. cDNA positions are according to transcript NM_022124.5. For none of the variants an effect on transcript splicing is predicted. V1-V11, variants in other (mouse) deafness genes than candidate gene *ATP2B2* as depicted in Fig S1; in house frequency is based on WES of ~20 000 individuals; GnomAD E EAS, allele frequency (%) in gnomAD, exomes of East-Asians; GnomAD E NFE, allele frequency (%) in gnomAD, exomes of non-Finnish Europeans; NA, not applicable. ^a^ Variant code used in Figure S1; ^b^ (Kircher et al. 2014); ^c^ (Kumar et al. 2009); ^d^ (Adzhubei et al. 2010); ^e^ (Schwarz et al. 2014); ^f^ Pathogenicty classification is based on guidelines from the Association for Clinical Genetic Science and the Dutch Society of Clinical Genetic Laboratory Specialists (Wallis et al. 2013); ^g^ Classification according to ClinVar. None of the variants were predicted to have an effect on splicing. Scores that meet the thresholds for pathogenicity are indicated in red. V, variant.

**Table S3 Rare *CDH23* variants in the index cases.**

| **Family code (index case)** | **Variant code^a^** | **Genome** | **cDNA** | **Protein** | **CADD**  **_PHREDD^b^** | **SIFT^c^** |  | **PPH2^d^** | **Mutation Taster^e^** | **Pathogenicity**  **Classification^f^** | **Pathogenicity class^g^** | **In house frequency (%)** | **gnomAD-E EAS (%)** | **gnomAD-E NFE (%)** |
| --- | --- | --- | --- | --- | --- | --- | --- | --- | --- | --- | --- | --- | --- | --- |
| W18-0138 (III.1) | VC1 | g.73491873A>G | c.3845A>G | p.Asn1282Ser | 14.17 | **0.00** |  | **0.796** | **Disease causing** | UV1/UV2 | UV1/UV2 | 0.694 | NA | 0.523 |
| W18-0111 (III.1) | VC2 | g.73377112G>A | c.1096G>A | p.Ala366Thr | **34.00** | **0.00** |  | **0.993** | **Disease causing** | UV1/UV2 | UV1/UV2 | 0.996 | NA | 0.902 |
|  | VC3 | g.73472494A>G | c.3293A>G | p.Asn1098Ser | **16.95** | **0.00** |  | 0.018 | **Disease causing** | UV1 | UV1/UV2 | 0.400 | NA | 0.386 |
| W17-4352 (III.2) | VC4 | g.73558192G>A | c.6911G>A | p.Arg2304Gln | **16.21** | **0.05** |  | 0.060 | Polymorphism | UV3 | UV1/UV23 | 0.016 | 0.026 | 0.004 |
| W17-0883 (III.1) | VC5 | g.73434968G>A | c.1514+35G>T | p.? | 0.14 | NA |  | NA | NA | NA | - | - | NA | - |
| W18-139 (III.3) | VC6 | g.73565712G>A | c.8022G>A | p.= | 11.12 | NA |  | NA | NA | UV1/UV3 | UV1-UV3 | 0.849 | NA | 0.851 |

In family W17-4352 GnomAD East Asian was also assessed due to ancestry. Genomic positions are according to GRCh37/hg19. cDNA positions are according to transcript NM_022124.5. For none of the variants an effect on transcript splicing is predicted. Amino acids changed in VC1-VC4 are located in cadherin domains 12, 4, 10 and 22, respectively. VC1-VC6, variants in *CDH23*, as listed in Figure S1, in house frequency is based on WES of ~20 000 individuals; GnomAD E EAS, allele frequency (%) in gnomAD, exomes of East-Asians; GnomAD E NFE, allele frequency (%) in gnomAD, exomes of non-Finnish Europeans; NA, not applicable. ^a^ Variant code used in Figure S1; ^b^ (Kircher et al. 2014); ^c^ (Kumar et al. 2009); ^d^ (Adzhubei et al. 2010); ^e^ (Schwarz et al. 2014); ^f^ Pathogenicty classification is based on guidelines from the Association for Clinical Genetic Science and the Dutch Society of Clinical Genetic Laboratory Specialists (Wallis et al. 2013); ^g^ Classification according to ClinVar. None of the variants were predicted to have an effect on splicing. Scores that meet the thresholds for pathogenicity are indicated in red.

**Table S4. Individual results of ABR measurements, vestibular history and vestibular testing**

| **Family** | **Subject (age)** | **Click-evoked ABR** | **Anamnesis and history of vestibular symptoms** | **Oculumotor testing** | **vHIT** | **Caloric measurement** | | | | |
| --- | --- | --- | --- | --- | --- | --- | --- | --- | --- | --- |
|  |  |  |  |  |  | **Warm** (°/s) (10-52)^a^ | | **Cold** (°/s) 7-31)^a^ | | **Conclusion** |
|  |  |  |  |  |  | AD | AS | AD | AS |  |
| W18-0111 | III.1 (11) | NT | no | N | N | 21 | 28 | 31 | 35 | N |
|  | II.3 (44) | NT | no | N | N | 29 | 30 | 18 | 11 | N |
| W18-0138 | III.1 (24) | NT | no | N | N | 19 | 11 | NT | NT | N |
| W17-0883 | III.1 (10) | symmetric, minor TWD related to HI | no | N | N | NT | NT | 24 | 24 | N |
|  | II.1 (31) | symmetric, minor TWD related to HI | no | N | N | 11 | 38 | 21 | 27 | N |
| W18-0139 | IV.2 (48)^c^ | symmetric, minor TWD related to HI | Balance complaints when standing or walking after cholesteatoma surgery with perilymph leak in left ear. | N | N | 20 | 4 | 16 | 3 | Right N |
|  | III.3 (68) | symmetric, minor TWD related to HI | Diagnosed with benign paroxysmal positional vertigo (BPPV) | N | N | 31 | 29 | 24 | 19 | N |

| **Family** | **Subject** | **Rotating chair/pendular chair^2^** | | | | | | | **cVEMP** | | | **oVEMP** | | |
| --- | --- | --- | --- | --- | --- | --- | --- | --- | --- | --- | --- | --- | --- | --- |
| (continued) |  | **Gain** (%) (33-72)^a^ | | **SPV** (^0^/s) (30-65)^a^ | | **Tau** (s) (11-26)^a^ | | **Conclusion** | **Threshold** (dBHL) (<100)^a^ | | **conclusion** | **Thresholds** (dBFL) (<140)^a^ | | **conclusion** |
|  |  | CW | CCW | CW | CCW | CW | CCW |  | AD | AS |  | AD | AS |  |
| W18-0111 | III.1 | 45 | 69 | 40 | 62 | 14 | 10 | normal | 100 | >100 | AD normal, AS no saccular function measured | 124 | 126 | Normal thresholds, no utricular lesions |
|  | II.3^b^ | 29 | 22 | 36 | 28 | NA | NA | normal | NT | NT | NA | NT | NT | NA |
| W18-0138 | III.1 | 73 | 59 | 66 | 53 | 22 | 16 | slightly hyperreactive to AD due to fear | >100 | >100 | no saccular function measured | >140 | >140 | No utricular function measured |
| W17-0883 | III.1 | 70 | 76 | 64 | 69 | 25 | 21 | slightly hyperreactive | 87 | 87 | Normal thresholds, no saccular lesions | NT | NT | NA |
|  | II.1 | 64 | 58 | 58 | 52 | 31 | 26 | slightly hyperreactive | 90 | 87 | Normal thresholds, no saccular lesions | NT | NT | NA |
| W18-0139 | IV.2^c^ | 31 | 46 | 30 | 47 | 11 | 10 | normal | 92 | >100 | AD normal, AS no saccular function measured | 135 | 132 | Normal thresholds, no utricular lesions |
|  | III.3 | 82 | 65 | 74 | 58 | 7 | 10 | CW slightly hyperreactive SPV and hypo-reactive Tau | 87 | 92 | Normal thresholds, no saccular lesions | 130 | 130 | Normal thresholds, no utricular lesions |

ABR, auditory brainstem response; vHIT, video head impulse test; cVEMP, cervical vestibular evoked myogenic potentials; oVEMP, ocular vestibular evoked myogenic potentials; °, degree; s, seconds; SPV, slow phase velocity; Tau, time constant; dBHL, decibel hearing level; dBFL, decibel force level; AD, right ear; AS, left ear; CW, clock-wise; CCW, counter clock-wise; TWD, total wave delay; N, normal; NT, not tested; NA, not applicable. ^a^, normal values at our institute; ^b^, subject W18-0111 II.3 had pendular chair testing instead of rotating chair testing. ^c^, only right ear results assessed.

**References**

Adzhubei IA, Schmidt S, Peshkin L, Ramensky VE, Gerasimova A, Bork P, Kondrashov AS, Sunyaev SR (2010) A method and server for predicting damaging missense mutations. Nat Methods 7: 248-9. doi: 10.1038/nmeth0410-248

Kircher M, Witten DM, Jain P, O'Roak BJ, Cooper GM, Shendure J (2014) A general framework for estimating the relative pathogenicity of human genetic variants. Nat Genet 46: 310-5. doi: 10.1038/ng.2892

Kumar P, Henikoff S, Ng PC (2009) Predicting the effects of coding non-synonymous variants on protein function using the SIFT algorithm. Nat Protoc 4: 1073-81. doi: 10.1038/nprot.2009.86

Schwarz JM, Cooper DN, Schuelke M, Seelow D (2014) MutationTaster2: mutation prediction for the deep-sequencing age. Nature Methods 11: 361-362. doi: 10.1038/nmeth.2890

Strehler EE, Zacharias DA (2001) Role of alternative splicing in generating isoform diversity among plasma membrane calcium pumps. Physiol Rev 81: 21-50. doi: 10.1152/physrev.2001.81.1.21

Wallis Y, Payne S, McAnulty C, Bodmer D, Sistermans E, Robertson K, Moore D, Abbs S, Deans Z, Devereau A (2013) Practice Guidelines for the Evaluation of Pathogenicity and the Reporting of Sequence Variants in Clinical Molecular Genetics. . ACGS/VGKL.

**URLs**

ClinVar, https://www.ncbi.nlm.nih.gov/clinvar/

GnomAD, http://gnoma d.broad institute.org/

Uniprot, http://www.uniprot.org/uniprot/Q01814

UCSC Genome browser, https://genome.ucsc.edu/

Ensemble Genome browser, <https://www.ensembl.org/index.html>
